# Multicenter evaluation of TB-SPRINT 59-Plex Beamedex®: accuracy and cost analysis

**DOI:** 10.1186/s12879-019-4646-3

**Published:** 2019-12-10

**Authors:** Regina Bones Barcellos, Isabela Neves de Almeida, Elisangela Costa da Silva, Harrison Magdinier Gomes, Lida Jouca de Assis Figueredo, Maria Laura Halon, Elis Regina Dalla Costa, Ícaro Rodrigues dos Santos, Maria Cláudia Vater, Suely Alves, Wânia da Silva Carvalho, Philip Suffys, Christophe Sola, Silvana Spíndola de Miranda, Maria Lucia Rossetti, Afrânio Kritski

**Affiliations:** 1 Centro de Desenvolvimento Científico e Tecnológico (CDCT), Secretaria Estadual da Saúde do Rio Grande do Sul (SES/RS), Porto Alegre, RS Brazil; 2 Laboratório de Pesquisa em Micobactérias, Faculdade de Medicina, Universidade Federal de Minas Gerais (UFMG), Belo Horizonte, MG Brazil; 3 Centro de Pesquisa em Tuberculose, Faculdade de Medicina, Universidade Federal do Rio de Janeiro (UFRJ), Rio de Janeiro, RJ Brazil; 4 Laboratório de Biologia Molecular Aplicada a Micobactérias, Instituto Oswaldo Cruz, Fundação Oswaldo Cruz (FIOCRUZ), Rio de Janeiro, RJ Brazil; 5 AstraZeneca do Brasil, Cotia, SP Brazil; 6 Faculdade de Farmácia (UFMG), Belo Horizonte, MG Brazil; 7 Institute for Integrative Biology of the Cell (I2BC), CEA, CNRS, Univ. Paris-Sud, Université Paris-Saclay, 91198 Gif-sur-Yvette cedex, France

**Keywords:** Tuberculosis, Resistance, TB-SPRINT, Genotype MTBDRplus, Isoniazid, Rifampicin

## Abstract

**Background:**

Molecular tests can allow the rapid detection of tuberculosis (TB) and multidrug-resistant TB (MDR-TB). TB-SPRINT 59-Plex Beamedex® is a microbead-based assay developed for the simultaneous spoligotyping and detection of MDR-TB. The accuracy and cost evaluation of new assays and technologies are of great importance for their routine use in clinics and in research laboratories. The aim of this study was to evaluate the performance of TB-SPRINT at three laboratory research centers in Brazil and calculate its mean cost (MC) and activity-based costing (ABC).

**Methods:**

TB-SPRINT data were compared with the phenotypic and genotypic profiles obtained using Bactec™ MGIT™ 960 system and Genotype® MTBDR*plus,* respectively.

**Results:**

Compared with MGIT, the accuracies of TB-SPRINT for the detection of rifampicin and isoniazid resistance ranged from 81 to 92% and 91.3 to 93.9%, respectively. Compared with MTBDR*plus,* the accuracies of TB-SPRINT for rifampicin and isoniazid were 99 and 94.2%, respectively. Moreover, the MC and ABC of TB-SPRINT were USD 127.78 and USD 109.94, respectively.

**Conclusion:**

TB-SPRINT showed good results for isoniazid and rifampicin resistance detection, but still needs improvement to achieve In Vitro Diagnostics standards.

## Background

Tuberculosis (TB) remains a major health problem worldwide. According to the World Health Organization (WHO), it is estimated that 10 million cases of TB occurred in 2017, causing 1.6 million deaths [1]. Multidrug Resistant tuberculosis (MDR-TB) is characterized by resistance to at least isoniazid (INH) and rifampicin (RIF), the two most important anti-TB drugs. Around 558,000 new cases of MDR-TB occurred worldwide in 2017 [1, 2].

In Brazil, about 82,676 TB cases were noted in 2016, out of which 1900 were estimated to be MDR-TB [1]. Brazil is one of the 30 high TB burden countries with cure rates that differ among the states. The median value of these cure rates is 71%, still far from the 85% goal of the WHO END TB Strategy [1].

The major challenge facing the success of TB treatment is patient acceptance of the treatment drug regimen along with a correct and early diagnosis of drug resistant strains. In most laboratories in Brazil and other countries with limited resources, smear microscopy examination is routinely used for TB diagnosis, while culture and drug susceptibility testing (DST) are mostly performed in reference laboratories as they require a biosafety level 3 laboratory [3, 4].

Molecular tests can provide rapid detection of TB and MDR-TB. The WHO has already endorsed and recommended some techniques for the rapid detection of TB and MDR-TB, such as the Genotype® MTBDR*plus* (MTBDR*plus*, HAIN Life Sciences, Nehren, Germany) and the Xpert® MTB/RIF (Cepheid, Sunnyvale, CA, USA) [1, 3, 5]. Recently, a consensus was reached regarding the importance to consider the test accuracy, time to produce a result, and costs incurred by a new diagnostic method, before its incorporation into the healthcare system [6–8].

In this study, a molecular technique called TB-SPRINT 59-Plex Beamedex® (TB-SPRINT, Beamedex, Orsay, France) was used to identify TB and MDR-TB. This technique is a microbead-based assay developed to run on Luminex® devices (Luminex Corp., Austin, TX, USA) for the simultaneous spoligotyping and detection of *rpoB*, *katG*, and *inhA* mutations associated with resistance to RIF and INH. It has also been successfully used in previous studies and performed well when compared to the MTBDR*plus* [9–11]*.* However, no data on its accuracy and costs have been gathered from a multicenter laboratory study considering all components of the cost chain. This includes the cost of the test (which is currently very low given its current marketing as a research use only (RUO) as well as an in vitro diagnostic (IVD) [9].

The aim of this study was to evaluate the performance of TB-SPRINT for MDR-TB detection at three laboratory research centers in Brazil and to evaluate its mean cost (MC) and activity-based costing (ABC).

## Methods

### Samples

This study was performed with a panel of 105 *Mycobacterium tuberculosis* (*Mtb*) convenience sample isolates selected randomly from the clinical collection of the Mycobacteria Research Laboratory of Medicine School of the Federal University of Minas Gerais (MRL/MS/UFMG). Using DST in the Bactec™MGIT™960 system (Becton Dickinson Microbiology System, Sparks, NV, USA), it was found that 69 of these isolates were RIF and INH sensitive and 36 were MDR [12]. The MTBDR*plus* assay was performed for all isolates of this panel according to the manufacturer’s instructions at MRL/MS/UFMG [13]. At Mycobacteria Research Laboratory/MS/UFMG, the DNA of *Mtb* isolates was extracted according to the protocol described by Dantas et al. (2015) [14]. After isolating the genomic DNA, it was aliquoted and sent blinded to each of the following sites: 1) Laboratory of Molecular Biology applied to Mycobacteria Research of Federal University of Rio de Janeiro (LMBMR/UFRJ) and 2) Laboratory of Molecular Biology applied to Mycobacteria of Oswaldo Cruz Foundation (LMBM/FIOCRUZ/RJ). Furthermore, all the sites performed the TB-SPRINT according to the manufacturer’s instructions [15].

### Tb-sprint

The high-throughput TB-SPRINT assay was performed and analyzed using the Luminex™ 200 flow cytometry device in the 3 different sites (UFMG, UFRJ, and FIOCRUZ) [11, 15]. In total, 59 probes were used, of which 43 were for spoligotyping and 16-plex format assay for RIF and INH detection of resistance-associated mutations (81 base pair rifampicin resistance determining region (RRDR) of the *rpoB* gene, *katG* codon 315, and *inhA* promoter region positions − 15 and − 8). The assays were performed as described previously [11].

### DNA sequencing

Target genes of both drugs were amplified as described elsewhere by Junior et al. (2014) [16] and submitted to DNA sequencing using Big Dye® Kit Terminator Cycle Sequencing (Applied Biosystems, Foster City, CA, USA). Capillary electrophoresis was performed with an automated genetic analyzer ABI Prism 3130xl (Applied Biosystems), following the manufacturer’s instructions. The DNA sequences obtained for each gene were analyzed using the Lasergene SeqMan software (DNASTAR©, Madison, USA) and compared with the reference sequences amplified from wild type H37Rv strain and sequences obtained at GenBank (MG995339, MG995338, CP023597, MG995071, MG995070) (National Center for Biotechnology Information – NCBI – https://www.ncbi.nlm.nih.gov/genbank/).

### Statistical analysis

Statistical analysis was performed using the Statistical Package for Social Sciences software v.21.0. The sensitivity (SE), specificity (SP), accuracy (A), kappa statistics (K), and McNemar discordance statistics were calculated based on the proportion of RIF and INH resistant and susceptible isolates identified by TB-SPRINT and MTBDR*plus* in comparison to the standard DST method.

The TB-SPRINT indeterminate results of all sites that did not have enough fluorescence reading value to confirm hybridization in the probes evaluated, were not included in the statistical analysis but were included in the cost analysis.

### Cost analysis

The cost study was developed in LMR/UFMG where all costs components were verified and not estimated [17]. The cost study was performed on this site, as it was the study coordinator site, and had all the data for the economic study duly available and approved by the Ethics Committee (CAAE -11821913.6.000.5257, CAAE – 0223.2412.7.1001.5149, DEPE/CH, protocol number 139/12).

The TB-SPRINT cost was calculated using the following two methods: the MC method and ABC method. In the MC method, the total cost of all cost components is divided by the quantity produced in a given period (total exams performed / in a month). Based on the LMR/UFMG routine, this study considered 15 positive cultures and analyzed them over a period of 1 month. Moreover, in the ABC method, the basic principle is to direct as many proportional and non-proportional costs as possible through cost drivers. This method is suitable for complex organizations, such as hospitals, where they consume resources in a very heterogeneous way [18].

The cost components to be considered to calculate the costs of the TB-SPRINT 59-Plex Beamedex® are the following: infrastructure; all equipment used like the Luminex or Bioplex 200®; the supplies necessary for the all steps of this method – DNA extraction - PCR, Hybridization – Luminex, and Bioplex 200® analysis including the calibration and validation kits; the Sheath fluid indispensable for the operation of these equipment; personal protective equipment (PPE); human resources; the cost of the sample collection and culture; and the values of maintenance of biosafety laboratories (BSL3).

## Results

### TB-SPRINT versus drug susceptibility testing

The DST identified 72.4% (69/105) samples as susceptible to RIF and INH, and 37.8% (36/105) as MDR-TB. The results for RIF and INH resistance detection obtained by TB-SPRINT*®* at the three research laboratories, and those obtained using MTBDRplus performed at UFMG, all in comparison to DST results (including sensitivity (SE), specificity (SP), positive predictive value (PPV), negative predictive value (NPV), accuracy (A), and concordance by kappa means value (K)), are displayed in Table 1. The TB-SPRINT indeterminate results from all sites stand for a mean of 22.8% of the tests.

**Table 1:** Compared TB-SPRINT 59-Plex Beamedex® results for rifampin and isoniazid resistance detection with BACTEC™ MGIT™ 960 system among the three sites evatuated, and Genotype®MTBDR*plus*between with BACTEC™ MGIT™ 960 system

				BACTEC™ MGIT™ 960							
				S	R	SE	SP	PPV	NPV	A	K*
TB-SPRINT	FIOCRUZ	RIF *n*=58	S	21	2	92.8	70	74.2	91.3	81	0.623
			R	9	26						
		INH *n*=69	S	32	4	88.5	94.1	93.9	88.9	91.3	0.826
			R	2	31						
	UFRJ	RIF *n*=57	S	47	3	70	100	100	94	94.7	0.794
			R	0	7						
		INH *n*=83	S	51	3	90	96.2	93.1	94.1	93.9	0.869
			R	2	27						
	UFMG	RIF *n*=78	S	45	2	93.1	91.8	87.9	95.7	92.3	0.838
			R	4	27						
		INH *n*=89	S	51	5	86.1	96.2	93.9	91	92.1	0.835
			R	5	28						
MTBDR*plus*		RIF *n*=105	S	69	1	97.2	100	100	98.5	99	0.979
			R	0	35						
		INH *n*=105	S	69	6	83.3	100	100	92	94.2	0.868
			R	0	30						

*RIF* rifampin, *INH* isoniazid, *S* sensitive, *R* resistant, *SE* sensitivity, *SP* specificity, *NPV* negative predictive value, *PPV* positive predictive value, *A* accuracy, *K* kappa value; *all kappa value showed *p* value < 0.001. Interpretation of Kappa value: < 0.20 = poor; 0,21–0,40 = weak; 0,41–0,60 = moderated; 0,61–0,80 = good; > 0,80 = very good

At the FIOCRUZ site, the TB SPRINT performance for RIF was as follows: SE of 92.8%, SP of 70.0%, PPV of 74.2%, NPV of 91.3%, A of 81.0%, and K = 0.623 (*p* < 0.001). Its performance for INH was: SE of 88.5%, SP of 94.1%, PPV of 93.9%, NPV of 88.9%, A of 91.3%, and K = 0.826 (*p* < 0.001).

At the UFRJ site, TB-SPRINT showed the following results for RIF: SE of 70%, SP of 100%, PPV of 100%, NPV of 94%, A of 94.7%, and K = 0.794 (*p* < 0.001). For INH, the results were as follows: SE of 90%, SP of 96.2%, PPV of 93.1%, NPV of 94.1%, A of 93.9%, and K = 0.869 (*p* < 0.001).

At the UFMG site, for RIF, TB-SPRINT showed the following results: SE of 93.1%, SP of 91.8%, PPV of 87.9%, NPV of 95.7%, A of 92.3%, and K = 0.838 (*p* < 0.001). For INH, the results were as follows: SE of 86.1%, SP of 96.2%, PPV of 93.9%, NPV of 91%, A of 92.1%, and K = 0.835 (*p* < 0.001).

McNemar’s discordance analysis did not reveal significance (all *p* values were > 0.05).

### MTBDRplus versus drug susceptibility testing

MTBDR*plus* detected no mutations for INH and RIF resistance in 69 strains (susceptible), 29 had mutations for both INH and RIF resistance (MDR), 1 strain had only a INH resistance related mutation, and 6 had only RIF resistant related mutations. Compared to DST, the MDTBDR*plus* for RIF had SE of 97.25, SP of 100%, PPV of 100%, NPV of 98.5%, A of 99%, and K = 0.979 (*p* < 0.001). The MDTBDR*plus* for INH had SE of 83.3%, SP of 100%, PPV of 100%, NPV of 92%, A of 94.3%, and K = 0.868 (*p* < 0.001). Moreover, McNemar’s discordance analysis did not reveal significant results (*p* ≤ 0.05).

### TB-SPRINT versus MTBDRplus

The concordance between TB-SPRINT and MTBDR*plus* is displayed in Table 2. When the assay was performed at FIOCRUZ, the K values for RIF and INH were 0.660 and 0.825, respectively. Moreover, the K values for RIF and INH were 0.794 and 0.864, respectively, at UFRJ and 0.838 and 0.828, respectively, at UFMG.

**Table 2:** Concordance values TB-SPRINT 59-Plex Beamedex® results for rifampin and isoniazid resistance detection with GenoType MTBDR*plus* among the three sites evatuated

		MTBDR*plus*		
		K		
TB-SPRINT	FIOCRUZ	RIF	0.660	*p* < 0.001
		INH	0.825	
	UFRJ	RIF	0.794	*p* < 0.001
		INH	0.864	
	UFMG	RIF	0.838	*p* < 0.001
		INH	0.828	

*RIF* rifampin, *INH* isoniazid, *K* kappa value. Interpretation of Kappa value: < 0.20 = poor; 0,21–0,40 = weak; 0,41–0,60 = moderated; 0,61–0,80 = good; > 0,80 = very good

### Costs analysis

The MC and ABC of the TB-SPRINT were USD 127.78 and USD 109.94, respectively. The values of the main equipment and supplies that impacted the cost chain are shown in Table 3. The ABC components of the TB-SPRINT are shown in Table 4, of which the supplies are the components with the greatest impacts, highlighting the values of the Luminex/Bioplex 200® reagents.

**Table 3:** Cost of the main equipment and inputs for TB-SPRINT 59-Plex Beamedex®

Equipment	Quantity	Unit Value	Mean Cost	ABC^a^
Luminex or Bioplex 200®	1	U$ 56.818	U$ 236.66	U$ 5.26
Termocycler	1	U$ 7.272	U$ 30.30	U$ 0.67
Refrigerated Centrifuged	1	U$ 6.089	U$ 25.37	U$ 0.56
Water purifier MilQ	1	U$ 4.454	U$ 22.72	U$ 0.50
Biological Safety Cabinet	1	U$ 38.461	U$ 3.20	U$ 0.48
Inputs	Quantity per exam	Unit Value	Mean Cost	ABC^a^
TB-SPRINT 59-Plex Beamedex®	1 kit for 100 reactions	U$ 265.15	U$ 39.77	U$ 2.65
Calibration Kit®	1 kit for six months	U$ 1.012	U$ 151.82	U$ 10.12
Validation Kit®	1kit for six months	U$ 2.476	U$ 371.47	U$ 24.76
Sheat Fluid®	1 kit for six months	U$ 276.81	U$ 41.52	U$ 2.77

^a^*ABC* Activity Based Cost

**Table 4:** Components costs and ABC of TB-SPRINT 59-Plex Beamedex®

TB-SPRINT 59-Plex Beamedex®	
Inputs	U$ 54.47
Assembled Procedures^a^	U$ 12.44
Human Resources	U$ 26.96
Equipaments and PM^b^	U$ 16.07
Total	U$ 109.94

Assembled Procedures^a^ = Collect and culture; ^b^PM = permanent material

### Sequencing profile

The sequencing results of the 105 isolates tested is displayed in Table 5. The 81-base pair *hotspot* of *rpoB* gene was evaluated for RIF resistance, and the *katG* codon 315 and the *inhA* − 15 promoter region were evaluated for INH resistance.

**Table 5:**
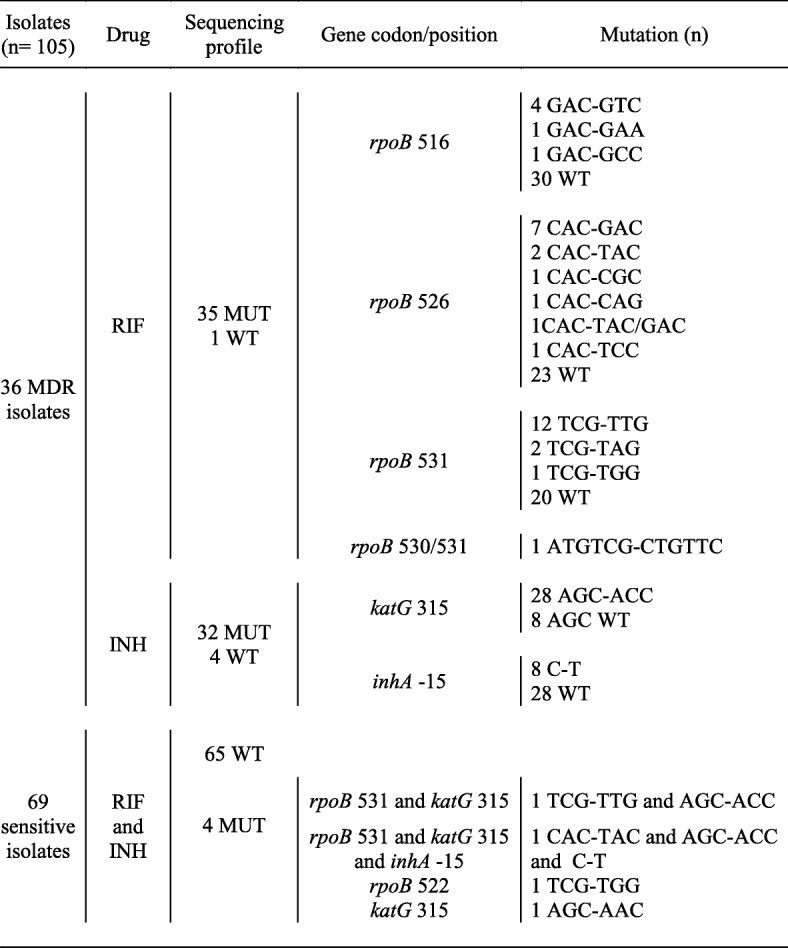
Sequencing profiles of the 105 isolates for *rpoB*, *katG* and *inhA* genes

*RIF* rifampim, *INH* isoniazid, *MUT* mutated, *WT* wild type

## Discussion

Overall, TB-SPRINT had results comparable to those of DST and MTBDRplus, presenting high agreement in values for INH and RIF resistance detection at all sites. Regarding accuracy, TB-SPRINT was able to detect resistance mutations in the RRDR region of *rpoB* gene, regarding RIF resistance, and in codon 315 of *katG* and in the position − 15 of the promoter region of *inhA* gene, regarding INH resistance. These results are close to the MTBDR*plus* accuracy values found in this study and previously described [19, 20]. When comparing the results between the sites, there was an important variation, which shows a reduced reproducibility of this molecular test.

A large number of indeterminate results in TB SPRINT were observed, mainly for the analysis of mutations in the *rpoB* gene. These results were similar to other studies observed in MTBDR*plus*, [21–23]. In previous studies, regarding evaluation of the frequency of indeterminate results of TB-SPRINT, only accuracy was evaluated [9, 11]. Most mutations conferring RIF resistance were identified in the 81-base pairs RRDR region of the *rpoB* gene, more frequently at codon 531, followed by codon 526, and codon 516. For INH resistance, most of the mutations were identified in the *katG* gene codon 315, followed by the *inhA* gene. These data are in agreement with the data described in the literature [11, 24]. Although most of the mutations found in the *rpoB* gene were the classic and most commonly observed ones (516 GAC-GTC, 526 CAC-GAC/TAC, and 531 TCG-TTG/TGG), other mutations were identified by sequencing in the evaluated MDR samples, which are not covered in the TB-SPRINT (Table 5). Such mutations can make the process of hybridization results analysis difficult to interpret, requiring more attention of the operator. Given the high number of indetermined TB-SPRINT outcomes observed in this study, and that the operator was blinded from the sequencing results at the time of the execution and data analysis, possible absence of signal in WT probes in resistant strains with other mutations were not detected, and the indetermined results, although described, were not evaluated in the statistic analysis. This is important to point out as every molecular result must be evaluated in conjunction with the patient’s clinical data, which could explain and avoid the false-negative results found [24].

The technique using Luminex devices allows the analysis of 96 samples at the same time, generating rapid results as recommended by the WHO [2]. Although excellent results were described previously, this was the first multicenter study that evaluated this test under routine conditions, with the same sample panel, changing only the operator and instruments [9, 11]. In this study, reduced reproducibility results have been observed, particularly for RIF, due to a high number of indeterminate results.

Differences in the laboratory structure, such as the fact that each site used its own reagents for PCR and hybridization, as TB-SPRINT did not provide PCR reagents in the kit at that time, may have contributed to the differences in reproducibility between sites and impacted the outcome of this test. Also, DNA extraction method is crucial to ensure good results, and as a limitation of this study, DNA extraction was performed only at UFMG and this material was distributed to the three sites, so the outcome regarding variability due to DNA extraction could not be accessed. Standardization of all laboratory flow and continuous personal training must be considered to achieve uniformity in the results. Despite the technical issue, the MC and ABC of TB-SPRINT (USD 127.78 and USD 109.94, respectively) are considerably higher than the average costs and ABC of MTBDR*plus* (USD 84.21 and USD 48.38, respectively) [25]. If the high number of repetitions of TB-SPRINT that would have been necessary was taken into account, these values would become even higher, causing this assay not suitable to be implemented in low and middle-income countries. Recently, after this study was performed, TB-SPRINT was improved, now it not only provides coupled beads but also dNTP, primers, and Taq Polymerase (www.beamedex.com) without significant extra costs.

The main components that increase the costs of TB-SPRINT are the equipment necessary supplies, where we highlight the high values of the calibration and validation kits indispensable for the use and maintenance of Luminex/Bioplex 200®, according to the manufacturer’s recommendation [26].

Other than the costs associated with TB-SPRINT, this kit also requires several steps to execute the test and does not provide all the required supplies. The supplies that are not provided by the kit and need to be acquired by the local laboratory include PCR plates, reading plates, adhesives, microtubes, and reagents for DNA extraction, PCR amplification, hybridization, and washing buffers [15]. Notwithstanding the cost increase, we observed a major variability between the laboratories that performed the assay, when evaluated under field conditions. This suggested that the protocol can be sensitive to variations in the instrument and reagents. Also, a protocol with many steps increases the amount of time dedicated by human resources and the need for equipment and materials. Although these are usually used in a molecular biology laboratory, they increase the values of both the MC and ABC. These data on TB-SPRINT MC and ABC, as well as the cost component analysis of this method, indicate that it may not be economically sustainable to incorporate TB-SPRINT into the drug resistance diagnostic routine of Brazilian public laboratories. The LMR/UFMG used as a model for the economic study, is a public laboratory that presents a reality very close to the other sites of this study and others public laboratories in Brazil, so is important highlight the necessity of laboratories perform the economic evaluations in loco in parallel to the performance evaluations, when incorporating new technologies.

Despite the fragile results for the identification of drug resistance in a routine laboratory, TB-SPRINT is an excellent method for performing spoligotyping as previously described by research laboratories as it gives great quality results in a short period of time [10, 11, 14].

The TB-SPRINT results were compared to DST by using MGIT, which was used as the standard method according to other studies evaluating the accuracy of this test for routine clinical detection of MDR-TB [9, 11]. Some strains present well-known resistance mutations, but are sensitive in DST. This event could occur due to the presence of heteroresistant strains, but it needs to be confirmed in the future studies, for instance retesting the strain for the DST together with minimum inhibitory concentration (MIC) determination, and whole sequencing genome [27].

This study presents some limitations: the relation of the clinical data of the patients with the results of the molecular tests was not performed, the cost-effectiveness was not evaluated, and the DNA extraction and the MTBDRplus assay were performed at one site. Given the WHO recommendations for rapid and reliable MDR strain detection, it is crucial to perform other studies to carry out laboratory validation and cost analysis, under field conditions, for any new molecular tests before their incorporation into the health system.

## Conclusions

In conclusion, the current version of TB-SPRINT 59-Plex Beamedex® may not be applicable yet in routine laboratories especially in locations of low resources, given the cost of maintenance and materials, however TB-SPRINT is also an unique assay, besides whole genome sequencing, that may provide interesting clues on MDR-TB transmission rates in a given setting. As TB-SPRINT not only describes the major drug-resistance mutations but also provides the genotyping (through spoligotyping). In this sense, TB-SPRINT could be well suited for large retrospective population-based multidrug resistance national survey.

TB-SPRINT 59-Plex Beamedex® showed good results for INH and RIF resistance identification, but still needs improvements to achieve IVD standards. The low cost of TB-SPRINT is also hampered by the high cost of purchasing a Luminex device and the costs associated with routine calibration-controls. The spread of the MagPix®, a 50-Plex LED based fluorescence imager (Luminex Corp, Austin, TX, USA), could significantly lower the routine cost of TB-SPRINT. Improved protocol and new cost analysis should be pursued for TB-SPRINT to be made suitable for routine MDR-TB molecular diagnostics.

## Data Availability

The datasets used and analyzed during the current study are available from the corresponding author on reasonable request.

## Abbreviations

A: Accuracy
ABC: Activity-based costing
DST: Drug sensitivity testing
INH: Isoniazid
IVD: In Vitro Diagnostic
K: Kappa means value
LMBM/FIOCRUZ/RJ: Laboratory of Molecular Biology applied to Mycobacteria of Oswaldo Cruz Foundation
LMBMR/UFRJ: Laboratory of Molecular Biology applied for Mycobacteria Research of Federal University of Rio de Janeiro
MC: Mean cost
MDR-TB: Multidrug-resistant TB
MRL/MS/UFMG: Mycobacteria Research Laboratory of Medicine School of the Federal University of Minas Gerais
*Mtb*: *Mycobacterium tuberculosis*
NCBI: National Center for Biotechnology Information
NPV: Negative predictive value
PCR: Polymerase chain reaction
PPE: Personal protective equipment
PPV: Positive predictive value
RIF: Rifampicin
RRDR: Rifampicin resistance determining region
RUO: Research use only
SE: Sensitivity
SP: Specificity (SP)
TB: Tuberculosis
TB-EFI: Ethambutol-fluoroquinolones-Injectable drugs
WHO: World Health Organization
